# Intensive Glucose Control Reduces the Risk Effect of *TRIB3, SMARCD3*, and *ATF6* Genetic Variation on Diabetic Vascular Complications

**DOI:** 10.3389/fphar.2018.01422

**Published:** 2018-12-11

**Authors:** Fazhong He, Yan Shu, Xingyu Wang, Xin Liu, Guojing Liu, Zhangren Chen, Zhenmin Wang, Ling Li, Rong Liu, Honghao Zhou, Heng Xu, Wei Zhang, Gan Zhou

**Affiliations:** ^1^Department of Clinical Pharmacology, Xiangya Hospital, Central South University, Changsha, China; ^2^Hunan Key Laboratory of Pharmacogenetics, Pharmacogenetics Research Institute – Institute of Clinical Pharmacology, Central South University, Changsha, China; ^3^National Clinical Research Center for Geriatrics, Xiangya Hospital, Central South University, Changsha, China; ^4^Department of Pharmaceutical Sciences, School of Pharmacy, University of Maryland, Baltimore, MD, United States; ^5^Beijing Hypertension League Institute, Beijing, China; ^6^Department of Laboratory Medicine, Precision Medicine Center, and Precision Medicine Key Laboratory of Sichuan Province, West China Hospital, Sichuan University, Chengdu, China; ^7^Sichuan and Collaborative Innovation Center, Chengdu, China

**Keywords:** type 2 diabetes, genetic variation, cardiovascular disease, individualized drug therapy, intensive glucose control

## Abstract

Type 2 diabetes mellitus is a complex disease. Our previous study revealed that *TRIB3* genetic variations were strongly associated with diabetic vascular complications, although *TRIB3* regulation pathways remain poorly understood. We used two extreme treatment groups from a 2 × 2 factorial randomized controlled trial to identify a positive association, which was further validated in patients receiving cross treatment to test the effect of genetic polymorphisms among the different treatment groups. A gene-centric score (GS)-weighted model including the three associated genetic variations *TRIB3* rs2295490, *ATF6* rs12086247, and *SMARCD3* rs58125572 was used. The results of the GS model indicated a 46% reduction in the risk of primary vascular complications in patients bearing more than two risk alleles [hazard ratio (HR) 0.54, 95% confidence interval (CI) 0.38–0.76, *p* < 0.001], following intensive glucose control treatment when compared with patients who received standard glucose control treatment. Furthermore, these patients benefited from active blood pressure-lowering treatment (HR 0.39, 95% CI 0.24–0.64, *p* < 0.001). However, no significant difference was observed between the two interventions in patients with fewer than two risk alleles (HR 1.09, 95% CI 0.86–1.39, *p* = 0.47). These results indicate that genetic variants in these three genes may be useful biomarkers for individualized drug therapy in diabetic patients.

## Introduction

Type 2 diabetes mellitus (T2DM) is a metabolic disease caused by a complex interplay of environmental and genetic variant risk factors (Xu et al., 2013). Results from previous studies have indicated that the clinical characteristics of T2DM are affected by common polygenic variants (Lango Allen et al., 2010). In particular, genome-wide association studies (GWAS) have established that single-nucleotide polymorphisms (SNPs) contribute to the susceptibility of T2DM and variations in fasting plasma glucose levels (FPG; Vaxillaire et al., 2014). However, the effects of these SNPs are modest, explaining only approximately 5.7% and 4.8% of the variability in T2DM and FPG, respectively (Morris et al., 2012; Scott et al., 2012). Currently, most known disease-associated SNPs (approximately 95%) are located in non-coding regions (Freedman et al., 2011). Delineating the mechanisms of these common genetic variations at intronic and/or intergenic regions with regard to disease susceptibility remains challenging. As an example, to date, all recognized T2DM-risk SNPs in the transcription factor 7-like 2 gene, which has the strongest association among all identified T2DM genetic loci, are intronic, and their mechanisms leading to T2DM have remained unclear for a decade (Jin, 2016).

The Encyclopedia of Deoxyribonucleic Acid (DNA) Elements (ENCODE) is an ongoing international cooperation project that has systematically listed functional elements, chromatin annotations, and variation annotations in the human genome, intuitively showing whether a SNP is located in any potential functional region, such as transcription factor binding sites, open chromatin regions, micro-ribonucleic acid (miRNA) and long non-coding RNA (lncRNA) transcription regions, miRNA target sites, and DNA methylation sites (ENCODE Project Consortium, 2012). Therefore, we believe that investigation of functional variants based on ENCODE may have great potential in facilitating the identification of valuable gene loci in disease and therapy (Mehta et al., 2013).

Patients with T2DM have a much higher risk of cardiovascular events relative to non-diabetic individuals (Williams et al., 2002). It is well supported by clinical trials that sustained hypotensive and antidiabetic treatment can significantly improve the outcomes of cardiovascular complications in patients with T2DM (Patel et al., 2007; Zoungas et al., 2014). Recently, the *TRIB3* gene was reported to play an integral role in insulin resistance-related T2DM and its cardiovascular complications (Prudente et al., 2012; Zhang et al., 2013). The Action in Diabetes and Vascular Disease: PreterAx and DiamicroN Modified-Release Controlled Evaluation (ADVANCE) project was designed to examine whether lowering blood pressure and intensively controlling glycemia in high-risk patients with T2DM could reduce the incidence of diabetic cardiovascular complications (Patel et al., 2007; Zoungas et al., 2014). Remarkably, the results of our previous pharmacogenomic study in ADVANCE (China centers) indicated that a mutation in the second exon (rs2295490, A > G) of *TRIB3* confers a different risk of primary vascular events in certain Chinese patients with T2DM. Specifically, patients with AG or GG genotypes experienced significantly fewer primary vascular events after receiving intensive glucose control treatment [aiming for a hemoglobin (Hb) A1c value of 6.5% or lower] as compared with patients receiving standard glucose treatment (patients who continued with their usual glucose control regimens). However, such a benefit was not observed in patients with the AA genotype (He et al., 2016). T2DM is a disease with complex traits, and a previous study suggested that core disease-related genes and genes outside of core pathways are sufficiently interconnected to affect its heritability (Boyle et al., 2017). Hence, we use the published bioinformatics tool (e.g., ENCODE) and database (e.g., STRING) to conduct this pharmacogenomic study in 1989 Chinese patients with T2DM who received blood pressure-lowering and glucose control treatments to determine whether any variants of candidate genes in the TRIB3 regulation pathway could provide a basis for an optimal treatment regimen to reduce cardiovascular complications in patients with T2DM.

## Patients and Methods

### Subjects

We performed a retrospective pharmacogenomic analysis using samples and data from a 2 × 2 factorial randomized controlled trial conducted in 61 centers in China over a follow-up period of 5 years. The original study protocol and details have been published previously (Patel et al., 2007; Group et al., 2008; He et al., 2016). Briefly, approval to conduct the trial was obtained from the ethics committee of each study center, and informed consent was obtained from all subjects (registration number NCT00145925). All methods were performed in accordance with the relevant guidelines and regulations. Patients (aged ≥ 55 years) with T2DM were randomly assigned (1:1) to receive either intensive glucose-lowering treatment (target HbA1c ≤ 6.5%) based on modified-release gliclazide (30–120 mg daily) or standard guideline-based therapy for glycemic control, as well as blood pressure-lowering treatment comprising perindopril and indapamide (initially 2.0 mg perindopril and 0.625 mg indapamide daily, increased to 4.0 mg perindopril and 1.25 mg indapamide daily after 3 months) or corresponding placebo. Participants and investigators were not blinded to the assignment of the glycemic control treatment but to that of the blood pressure-lowering treatment. During the follow-up period, any other antidiabetic, antihypertensive, antiplatelet, and antilipemic agents required were also administered according to the protocol.

### Candidate Genes and SNP Selection

**FIGURE 1:**
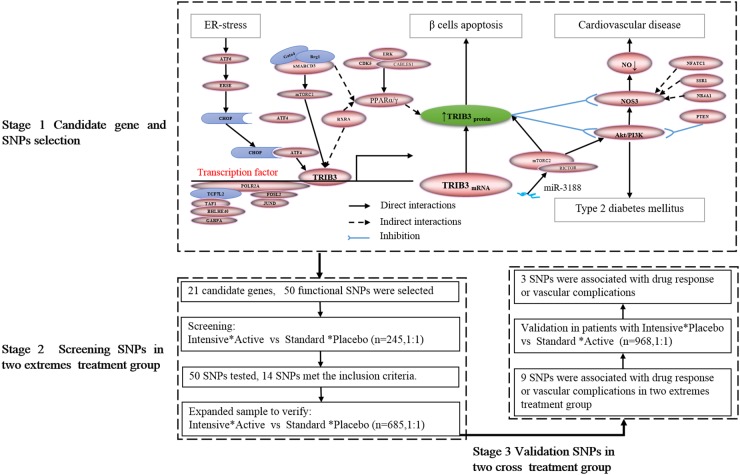
Candidate biomarker selection and pipeline of study protocol.

We used bioinformatic databases [STRING^1^, score > 0.90, top 20, and KEGG (glucose and lipid metabolic pathways^2^)] and searched the literature to predict candidate genes in the TRIB3 regulation pathways, as summarized in Stage 1 of Figure 1. The potential function of a SNP in or near the candidate gene was assessed using the ENCODE database^3^. Candidate SNPs with minor allele frequency ≥ 5% and pairwise linkage disequilibrium *r*^2^ < 0.30 within the same and adjacent genes [1000 Genomes phase 3 Han Chinese in Beijing (CHB) and Han Chinese South (CHS) data] were included.

### Study Design and Genotyping

A three-step association study were conducted (Figure 1). Selected SNPs were first screened to identify any significant association between a SNP and drug response/clinical outcomes in the two treatment cohorts (intensive glycemic control plus active blood pressure-lowering treatment, versus standard glycemic control plus matched placebo for blood pressure lowering, Stage 1). Next, expanded cohorts receiving the same treatments were used to replicate the significant association (Stage 2). Lastly, in Stage 3, the replicated association in Stage 2 was examined using cohorts receiving cross treatments (intensive glycemic control plus matched placebo for blood pressure lowering, versus standard glycemic control plus active blood pressure-lowering treatment) and compared to that in Stages 1 and 2. Genomic DNA was extracted from peripheral venous blood using a DNeasy Blood & Tissue Kit (QIAGEN, China). Candidate SNPs were genotyped by Bioyong Technologies Inc. using a Sequenom MassARRAY^®^SNP system for all patients, 5% of whom were randomly selected for genotype validation via Sanger sequencing.

### Primary Endpoint Measurements

Mean glycated Hb levels, systolic blood pressure (SBP), and diastolic blood pressure (DBP) measured during the follow-up period were used to assess drug response across the treatment groups. Major Macrovascular and microvascular events, as well as second primary endpoints have been defined in previous studies (Patel et al., 2007; Group et al., 2008). Briefly, major macrovascular events included death from cardiovascular causes, nonfatal myocardial infarction, or nonfatal stroke. Major Microvascular events included new or worsening renal or diabetic eye diseases. Second endpoints include coronary events, heart disease, cerebrovascular events, visual deterioration, new or worsening microalbuminuria (defined as urinary albumin and creatinine levels between 30 and 300 μg/mg), death from any other causes, neuropathy, hypoglycemia (recorded as fasting blood glucose level < 2.8 mmol/L), and vascular disease-related hospitalization. Relations between SNPs and major or second clinical outcomes were considered together and separately.

### Statistical Analysis

The genotyping and clinical data were summarized and analyzed via tests such as the Hardy–Weinberg equilibrium test, linkage disequilibrium test, as well as linear and logistic regression analyses with adjustment for baseline of body mass index, sex, age, drug dosage, duration of T2DM, combined medication, and other biomarkers (i.e., potassium concentration and low-density lipoprotein levels) using PLINK v1.9^4^. SNPs that met the inclusion criteria were further confirmed via mixed linear regression and Cox proportional hazards models with adjustment for the baselines using SAS software (version 9.4, SAS Institute). For participants who met more than one endpoint, the time of occurrence of the first relevant endpoint was used to calculate the survival time. We used a full range of genotypic models (additive, dominant, and recessive genetic models) to test any differences in categorical cluster variables and quantitative variables among the four treatment groups. The reduction in relative risk was calculated as (1-hazard ratio) × 100%. The quantitative data described in the text and figures are presented as means ± standard deviation (SD) or mean (inter-quartile range, IQR) and percentages for categorical data. Finally, associated SNPs were assigned a weighted gene-centric score (GS) by assigning each allele equal weight (Busch et al., 2017). The interactions between the subgroups and the treatments were then analyzed as described above for that between genotypes and treatments. A calculated two-tailed *p*-value of <5% was considered statistically significant.

## Results

### Baseline Characteristics, Genotyping Results and Outline of Major Findings

Peripheral venous blood samples were obtained from 1989 Chinese patients with T2DM across 61 clinical trial centers. Ninety-one (4.6%) DNA samples did not qualify for genetic testing. Of all the participants, 94.6% (1796/1898) successfully completed the trial as scheduled with an average follow-up period of 4.8 years. The baseline characteristics between the two treatment axes (glycemic control and blood pressure lowering) did not exhibit any significant differences (Table 1). A total of 50 SNP loci in 21 genes (Supplementary Table S1) were successfully identified in our study. According to our study protocol (Figure 1), the association was replicated for nine SNPs (Supplementary Figure S2), and genetic variations in three SNPs (*TRIB3* rs2295490, *ATF6* rs12086247, and *SMARCD3* rs58125572) were significant associated with diabetic vascular complications after glucose control and/or blood pressure-lowering intervention. However, these genetic polymorphisms did not affect drug response with regard to HbA1c%, SBP, and DBP (data not shown).

**Table 1:** Clinical characteristics of patients at baseline according to glucose control cohort and blood pressure lowering cohort^∗^.

Characteristic	Glucose control cohort		Blood pressure lowering cohort	
		
	Intensive (*N* = 945)	Standard (*N* = 953)	Active (*N* = 941)	Placebo (*N* = 957)
Male sex, n (%)	472(48.2)	507(51.8)	469(47.9)	510(52.1)
Age(yr), mean (SD)	65 ± 6	65 ± 6	65 ± 6	65 ± 6
Age when diabetes first diagnosed(yr), mean (SD)	57 ± 8	57 ± 8	57 ± 8	57 ± 8
Duration of diabetes, mean (IQR)	8(3–11)	8(3–11)	8(3–11)	8(3–11)
**Blood-pressure assessment**		
Systolic blood pressure(mmHg), mean (SD)	139.0 ± 21.1	140.8 ± 21.1	139.9 ± 21.3	139.9 ± 21.0
Diastolic blood pressure(mmHg), mean (SD)	78.5 ± 11.1	78.6 ± 11.0	78.3 ± 11.0	78.7 ± 11.1
**Blood-glucose assessment**		
Glycated hemoglobin (%), mean (SD)	7.7 ± 1.8	7.7 ± 1.7	7.7 ± 1.8	7.8 ± 1.7
Fasting blood glucose (mmol/L), mean (SD)	8.8 ± 3.0	8.6 ± 2.9	8.8 ± 3.1	8.6 ± 2.8
**Other major risk factors assessment**		
BMI (kg/m^2^), mean (SD)	25.4 ± 3.2	25.2 ± 3.1	25.2 ± 3.2	5.4 ± 3.1
History of major macrovascular disease, n (%)	321(34.0)	312(32.7)	308(32.7)	325(34.0)
History of major microvascular disease, n (%)	129(13.7)	139(14.6)	134(14.2)	134(14.0)
Current smoking, n (%)	219(23.2)	225(23.6)	214(22.7)	230(24.0)
Serum creatinine (umol/l), mean (SD)	79.8 ± 24.5	81.4 ± 32.5	79.5 ± 23.7	81.7 ± 33.1
Urinary albumin: creatinine(mg/mmol), median (IQR)	19.7(1.1–7.6)	22.8(1.1–6.9)	19.7(1.1–7.6)	20.3(1.1–6.5)
Total cholesterol(mmol/l), mean (SD)	5.4 ± 1.3	5.3 ± 1.2	5.4 ± 1.3	5.3 ± 1.2
High-density lipoprotein(mmol/l), mean (SD)	1.3 ± 0.4	1.3 ± 0.4	1.3 ± 0.4	1.3 ± 0.4
Low-density lipoprotein(mmol/l), mean (SD)	3.3 ± 1.0	3.2 ± 1.0	3.2 ± 1.0	3.2 ± 1.0
Triglyceride(mmol/l), mean (SD)	2.0 ± 1.8	2.0 ± 1.7	2.0 ± 1.8	2.0 ± 1.7
**Use of hypoglycemic agents**		
Gliclazide, n (%)	38(4.0)	37(3.9)	35(3.7)	40(4.2)
Other sulfonylurea, n (%)	642(67.9)	666(69.9)	656(69.9)	652(68.0)
Metformin, n (%)	588(62.2)	595(62.4)	602(64.1)	581(60.6)
Insulin, n (%)	20(2.1)	21(2.2)	22(2.3)	19(2.0)
Other antidiabetic agents, n (%)	264(27.9)	239(25.1)	253(26.9)	250(26.1)
**Use of antihypertensive agents**		
Perindopril, n (%)	20(2.1)	30(3.1)	24(2.6)	26(2.7)
Other ACE-I, n (%)	189(20.0)	189(19.8)	179(19.1)	199(20.8)
ARB, n (%)	15(1.6)	19(2.0)	19(2.0)	15(1.6)
B-blockers, n (%)	108(11.4)	93(9.8)	102(10.9)	99(10.3)
Diuretics, n (%)	105(11.1)	109(11.4)	113(12.0)	101(10.6)
Calcium antagonists, n (%)	320(33.9)	325(34.1)	320(34.1)	325(33.9)
Other BP lowering drug, n (%)	200(21.2)	204(21.4)	200(21.3)	204(21.3)
**Use of lipid-lowering and antiplatelet agents**		
Lipid-lowering agents(statins)	150(15.9)	152(15.9)	144(15.3)	158(16.5)
Antiplatelet agents (aspirin), n (%)	442(46.8)	415(43.5)	422(44.8)	435(45.5)


*^∗^All *P*-values > 0.05 between the group, data were not shown in the table.*

### SNPs in Response to Glycemic Control and Blood Pressure-Lowering Treatment

**FIGURE 2:**
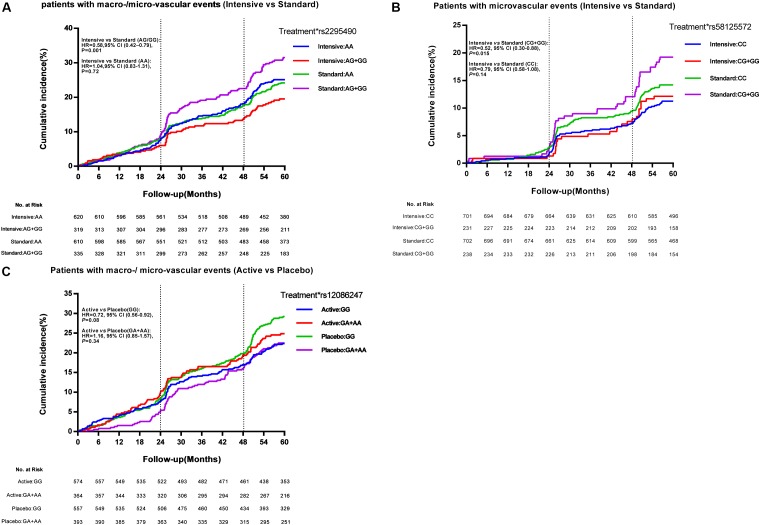
Effect of *TRIB3* (rs2295490), *SMARCD3* (rs58125572), and *ATF6* (rs12086247) genetic variation on primary cardiovascular events. Panels **(A–C)** depict the positive effect of *TRIB3, SMARCD3*, and *ATF6* genetic variants on the cumulative incidence of major vascular events according to glucose control or blood pressure-lowering treatment. The vertical dotted line represents additional data on microvascular events collected at the 24-month and 48-month study visits. The time to event was recorded as the date of visit. At month 57, 99% of events had occurred and the curves were truncated. The effects of the genetic variations were analyzed using unadjusted Cox proportional hazards models.

For the glucose control arm, as shown in Figure 2A, the *TRIB3* (rs2295490) AG/GG genotypes were found to reduce primary vascular events in patients who received the intensive glucose treatment as compared to those receiving the standard glucose treatment [hazard ratio (HR), 0.58; 95% confidence interval (CI), 0.42–0.79; *p* = 0.001]. Interestingly, as shown in Figure 2B, the relative risk of major microvascular events was significantly reduced in patients with the CG or GG genotype of SMARCD3 (rs58125572) genetic variation, when compared the intensive with the standard glycemic control treatment (HR, 0.52; 95% CI 0.30–0.88; *p* = 0.015). This was not observed in patients with the CC genotype (HR, 0.79; 95% CI 0.58–1.08; *p* = 0.14). However, we were unable to replicate any effects caused by the other six SNPs for which an association with clinical outcomes was suggested in our Stage 2 analysis (Supplementary Figure S1). For the blood pressure-lowering intervention arm, the *ATF6* (rs12086247, G > A) GG genotype was significantly associated with a reduction in microvascular events (HR, 0.71; 95% CI 0.51–1.00; *p* = 0.046; Supplementary Figure S2), but not in major macro-/micro-vascular events (HR, 0.72; 95% CI 0.56–0.92; *p* = 0.08; Figure 2C) when comparing patients receiving the active blood pressure treatment as compared to those receiving the placebo. However, such a reduction was not observed among patients with the GA/AA genotypes, and the other seven SNPs seemed to be irrelevant to clinical outcomes related to the blood pressure-lowering treatment (Supplementary Figure S2).

### Gene-Centric Score (GS) Analysis of *TRIB3, SMARCD3*, and *ATF6*

**FIGURE 3:**
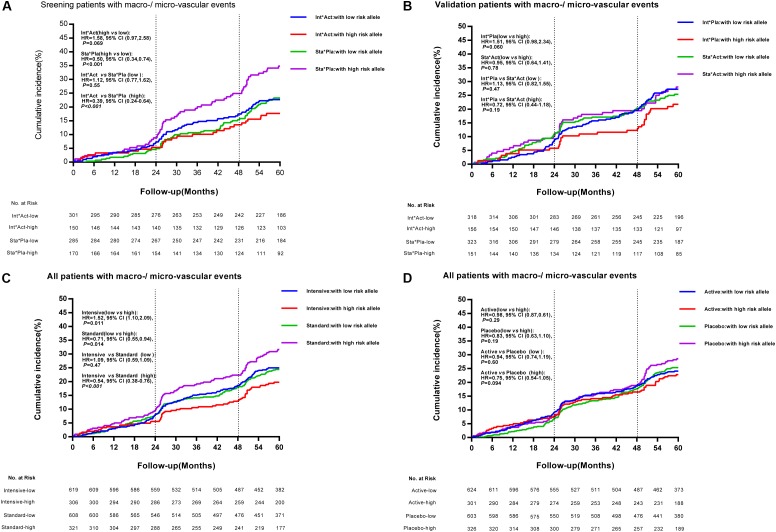
Cumulative incidence of combined major macrovascular and microvascular events among individuals with high- and low-risk alleles according to treatment strategies. Panel **(A)** depicts the cumulative incidence of combined major macrovascular and microvascular events in the screening stage (patients receiving combined intensive [Int] and active [Act] treatment, versus patients receiving combined standard [Sta] and matched placebo [Pla] treatment) among individuals with high-risk and low-risk alleles. Panel **(B)** represents the cumulative incidence of combined major macrovascular and microvascular events in the validation cohorts (patients receiving combined Int and Pla treatment, versus those receiving combined Act and Sta intervention) among individuals with high-risk and low-risk alleles. Panels **(C,D)** show the cumulative incidence of combined major macrovascular and microvascular events among individuals with high-risk and low-risk alleles according to glucose control and blood pressure-lowering treatment.

We defined the *TRIB3* (rs2295490, A > G) G allele, the *SMARCD3* (rs58125572, C > G) G allele, and the *ATF6* (G > A) G allele as risk alleles for vascular complications in patients with T2DM. Patients having zero to two risk alleles were defined as the low-risk subgroup, and those with three or more risk alleles as the high-risk subgroup. Using the GS analysis model, for glucose control arm, we observed that the relative risk of major macrovascular and microvascular events was significantly reduced by 46% following intensive glycemic control treatment as compared with the standard glycemic control treatment in patients in the high-risk group (≥3 risk alleles; HR, 0.54; 95% CI 0.38–0.76; *p* < 0.001). However, no significant differences were observed between the intensive and the standard glycemic control treatments in patients with fewer than two risk alleles (Figure 3C). Moreover, as shown in Figure 3A, compared with the diabetic patients receiving the standard glycemic control treatment and the matched placebo blood pressure-lowering treatment, those with three or more risk alleles who received the intensive glycemic control treatment and the active blood pressure-lowering treatment had a significantly reduced risk of major macrovascular events combined with microvascular events (HR, 0.39; 95% CI 0.24–0.64; *p* < 0.001). This was not observed in patients with fewer than two risk alleles (HR, 1.12; 95% CI 0.77–1.62; *p* = 0.55). In addition, as shown in Figure 3B, we did not observe any differences in the incidence of major macro-/micro-vascular events between high-risk and low-risk patients with the cross treatment (intensive glucose control treatment and matched placebo blood pressure-lowering treatment, or standard glucose control treatment and active blood pressure-lowering treatment). These results, as compared with those receiving standard glucose control treatment and the placebo for blood pressure-lowering treatment, suggest high-risk patients benefited from the intensive glucose control treatment and the active blood pressure-lowering treatment. For blood pressure control arm, no significant differences in major macrovascular events, microvascular events, and any other secondary clinical outcomes were observed between patients with three or more risk alleles and those with two or fewer (Figure 3D). The time-weighted mean HbA1c %, SBP, and DBP were similar between the two patient populations with different genotypes who received the antihypertensive treatment (data not shown).

## Discussion

**FIGURE 4:**
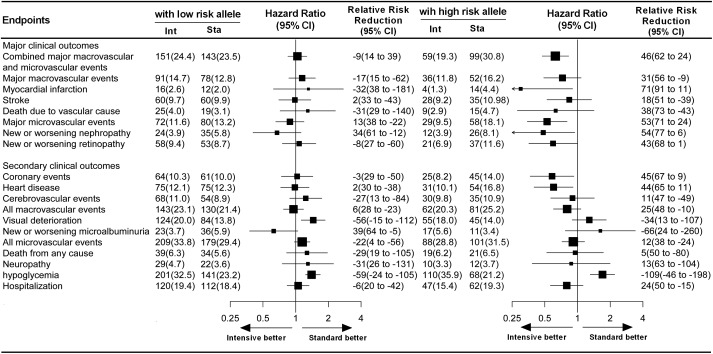
Effect of glucose treatment on primary and secondary endpoints among patients with high-risk and low-risk alleles. For each sub-endpoint, black squares represent point estimates, with the area of the square proportional to the number of events, and horizontal lines represent the 95% confidence interval. The hazard ratios and relative risk reductions are provided for intensive glucose control as compared with standard glucose control in patients with high-risk and low-risk alleles.

The ADVANCE project was designed to examine the effects of blood pressure-lowering treatment and intensive glycemic control on the occurrence of primary vascular events in hypertensive or normotensive patients with T2DM. After a 5-year follow-up period, the relative risk of primary vascular endpoints was reduced by 9 and 10% in patients receiving the active blood pressure-lowering treatment and in those receiving the intensive glycemic control treatment, respectively, when compared with their matched controls (Patel et al., 2007; Zoungas et al., 2014). Originally, this study was designed to determine the contribution of genetic variation to these effects in response to different drug treatments. In our study cohort, however, apart from modified-release gliclazide and fixed-dose perindopril and indapamide tablets, which were regularly used in the pre-specified intervention group, a wide range of other drugs was also irregularly used to achieve therapeutic goals in patients with T2DM. Therefore, the aim of our study, in the context of clinical practice, was to focus on exploring the relationship between genetic variants and different glucose or blood pressure homeostasis status among the 2 × 2 factorial treatment strategies in patients with T2DM. Our data showed that among patients with multiple high risk alleles likely leading to increased TRIB3 function, the intensive glycemic control treatment reduced the relative risk of primary vascular endpoints by 46% (Figures 3C, 4) as compared with the standard glycemic control treatment. However, such benefits in response to intensive glycemic control treatment were insignificant among patients with less genetic changes in TRIB3 gene.

Through a SNP selection strategy based on the ENCODE genome database, we identified two novel SNPs associated with drug treatment protocol-related vascular complications in patients with T2DM (as shown in Figures 2B,C). One SNP (rs58125572) introduced a DNA methylation site at chromosome 7 q36.1, which is the locus for a gene called switching defective/sucrose non-fermenting (SWI/SNF) related, matrix-associated, actin-dependent regulator of chromatin subfamily D, member 3 (*SMARCD3*). SMARCD3 is a core component of the SWI/SNF protein complex, which uses the energy generated by ATP hydrolysis to alter DNA–histone interactions, thereby facilitating the function of transcriptional activators involved in glycolytic metabolism via its induction of DEP domain-containing mTOR-interacting protein (DEPTOR) expression and protein kinase B (Akt) activation, and thus improving insulin resistance and lowering blood glucose (Peterson and Tamkun, 1995; Meng et al., 2013). No previous data are available with regard to the clinical relevance of this SNP in response to drug treatment and disease. The other SNP (rs12086247) located in activating transcription factor 6 (ATF6) intron region, can result in the removal of a DNA methylation site at chr1:161764793-161764793. Available data shows ATF6 was involved in unfolded protein responses related to reticulum stress via the direct induction of antioxidant proteins, and plays an important role in protecting myocardial ischemia injury (Jin et al., 2017). Three SNPs (rs4579731, rs10918215, and rs13401) in *ATF6* are reported to be significantly associated with FPG levels in Dutch Caucasian individuals. However, this association is not observed in a Chinese population, probably due to differences in the study cohort and the mutation frequency of the three SNPs (Meex et al., 2007; Hu et al., 2011). It is worth noting that TRIB3 acts as a brake on insulin-mediated signaling pathways, affecting many aspects of cell function via protein interactions (Eyers et al., 2017). The protein–protein interaction between TRIB3 and SMARCD3 was predicted by the STRING database. Studies revealed that SMARCD3, β-catenin, and TRIB3 appear to act as positive modulators of Akt (Klaus et al., 2012; Ozcan et al., 2013). However, there is no direct evidence currently supporting the regulation of TRIB3 by SMARCD3 or vice-versa. Lale et al. reported the clear regulation of TRIB3 by ATF6 and elucidated the crucial function of these two proteins in the development of metabolic diseases (Zhou et al., 2016).

To our knowledge, pharmacogenomic findings with respect to antidiabetic drug response or adverse drug reactions have yet to be translated into clinical application (Staiger et al., 2009). This may be related to the polygenic traits involved in both drug response and T2DM (Zoungas et al., 2010). In the present study, we observed that only patients with high-risk alleles receiving intensive glycemic control treatment experienced a significant reduction in major vascular or microvascular events, while intensive glycemic control treatment did not confer cardiovascular protection in low-risk allele carriers (Figure 4). These results suggest that not all diabetic patients need intensive glucose control, and the treatment strategy selected for target glucose and blood pressure status achieved need according to their genetic variants. Severe hypoglycemia has been reported to be strongly related to the incidence of macrovascular and microvascular events (Zoungas et al., 2010). Moreover, in our study cohorts, the mean age of participants was 65 years. Thorpe et al. (2015) reported that older diabetic patients experiencing strong glycemic control (HbA1c < 7.0%) did not experience long-term benefits but rather an increased number of adverse events. However, our data suggest that intensive glycemic control and active blood pressure-lowering treatment are necessary for diabetic patients with high-risk alleles for *TRIB3, SMARCD3*, and *ATF6*.

## Conclusion

*TRIB3* rs2295490, *ATF6* rs12086247, and *SMARCD3* rs58125572 genetic variants are associated with specific treatment protocols and with the incidence of diabetic vascular complications. Our results indicate that genetic variants in these three genes may be useful biomarkers for individualized drug therapy in diabetic patients.

## Author Contributions

FH wrote the manuscript. FH, WZ and GZ designed the study. FH, GL, ZC, ZW and LL performed the research. FH and RL analyzed the data. FH and YS interpreted and revised the manuscript. HZ, XL, XW, HX, WZ and GZ were responsible for data management and contributed reagents and materials to the study.

## Conflict of Interest Statement

The authors declare that the research was conducted in the absence of any commercial or financial relationships that could be construed as a potential conflict of interest.

## Footnotes

1 http://string-db.org/

2 http://www.genome.jp/kegg/pathway.html

3 http://www.genome.ucsc.edu/ENCODE/

4 http://www.cog-genomics.org/plink/1.9/
