# Supplementary material for: Intensive Glucose Control Reduces the Risk Effect of TRIB3, SMARCD3, and ATF6 Genetic Variation on Diabetic Vascular Complications
Source: Front Pharmacol. 2018 Dec 11;9:1422. doi: 10.3389/fphar.2018.01422 (PMC6297143; doi:10.3389/fphar.2018.01422)
Supplement: Supplementary file 1 [file Data_Sheet_1.pdf]

Table S1 List of candidate genes and selected SNPs

No.	SNP	chr	MAF	P_HWD	gene name	Function prediction
1	rs12985477	chr19	0.46	0.62	AKT2	lncRAN
2	rs11880261	chr19	0.37	0.44	AKT2	lncRAN
3	rs35920692	chr22	0.17	0.83	ATF4	TFBS
4	rs140663563	chr11	0.18	0.68	ATF4P4	lncRAN
5	rs74337724	chr11	0.16	1.00	ATF4P4	lncRAN
6	rs73361094	chr14	0.02	1.00	ATF4P4	lncRAN
7	rs2499847	chr1	0.40	1.00	ATF6	DNAase clusters
8	rs12032568	chr1	0.29	0.88	ATF6	lncRAN
9	rs12086247	chr1	0.26	0.20	ATF6	meDIP CpG
10	rs3767635	chr1	0.05	1.00	ATF6	Open chromatin regions
11	rs908078	chr3	0.21	0.35	BHLHE40	lncRAN
12	rs1104976	chr3	0.18	0.41	BHLHE40	predict promoter
13	rs2164029	chr18	0.45	0.27	CABLES1	open chromatin regions
14	rs77391796	chr18	0.16	1.00	CABLES1	lncRAN
15	rs2069452	chr7	0.44	0.62	CDK5	TFBS/predict promoter
16	rs1549759	chr7	0.03	1.00	CDK5	TFBS
17	rs10984901	chr9	0.49	0.15	CDK5RAP2	lncRAN
18	rs3780672	chr9	0.20	1.00	CDK5RAP2	lncRAN
19	rs7144658	chr14	0.14	0.44	FOXA1	lncRAN
20	rs1057536	chr14	0.12	0.14	FOXA1	predict promoter
21	rs4817056	chr21	0.05	1.00	GABPA	TFBS
22	rs7247237	chr19	0.39	0.37	JUND	UTR-3/MIR3188
23	rs1057079	chr1	0.20	1.00	MTOR	mTOR-AS1
24	rs2275526	chr1	0.08	0.41	MTOR	lncRAN
25	rs17027478	chr1	0.06	0.61	MTOR	CPG island
26	rs8095551	chr18	0.22	0.48	NFATC1	TFBS
27	rs11661539	chr18	0.02	1.00	NFATC1	miR-143 regulatory site
28	rs66727674	chr7	0.23	0.86	NOS3	lncRAN
29	rs1283155	chr12	0.40	0.90	NR4A1	lncRAN
30	rs71541942	chr17	0.34	0.59	POLR2A	open chromatin regions
31	rs881740	chr22	0.14	1.00	PPARA	lncRAN
32	rs10427609	chr22	0.01	1.00	PPARA	nearGene-3
33	rs4842194	chr9	0.34	0.42	RXRA	lncRAN
34	rs62576340	chr9	0.10	0.74	RXRA	TFBS
35	rs3750546	chr9	0.06	1.00	RXRA	TFBS
36	rs1536478	chr9	0.34	0.28	RXRA-COL5A1	lncRAN
37	rs3118590	chr9	0.21	1.00	RXRA-COL5A1	lncRAN
38	rs58125572	chr7	0.12	1.00	SMARCD3	DNAmehylation,lncRNA
39	rs219229	chr7	0.09	0.48	SMARCD3	lncRAN
40	rs3838329	chr7	0.09	0.47	SMARCD3	Enhancer

Table_S1 Continue

No.	SNP	chr	MAF	P_HWD	gene name	Function prediction
41	rs113745074	chr7	0.01	1.00	SMARCD3	CPG island
42	rs35746260	chr7	0.01	1.00	SMARCD3	CPG island
43	rs9505118	chr6	0.39	0.37	SSR1	lncRAN
44	rs2274210	chr6	0.33	0.21	SSR1	DNAase clusters/ncRNA
45	rs16827760	chr3	0.05	1.00	TBL1XR1	lncRAN
46	rs10788814	chr1	0.04	1.00	THEM4	lncRAN
47	rs11470129	chr20	0.35	0.69	TRIB3	predict promoter
48	rs4815567	chr20	0.25	0.41	TRIB3	lncRAN
49	rs2295490	chr20	0.19	0.70	TRIB3	missense_variant
50	rs11185665	chr9	0.04	1.00	WDR5-RXRA	lncRAN

Figure S1 Effect of genetic variants on major macrovascular, microvascular events or both according to glucose intervention treatment.

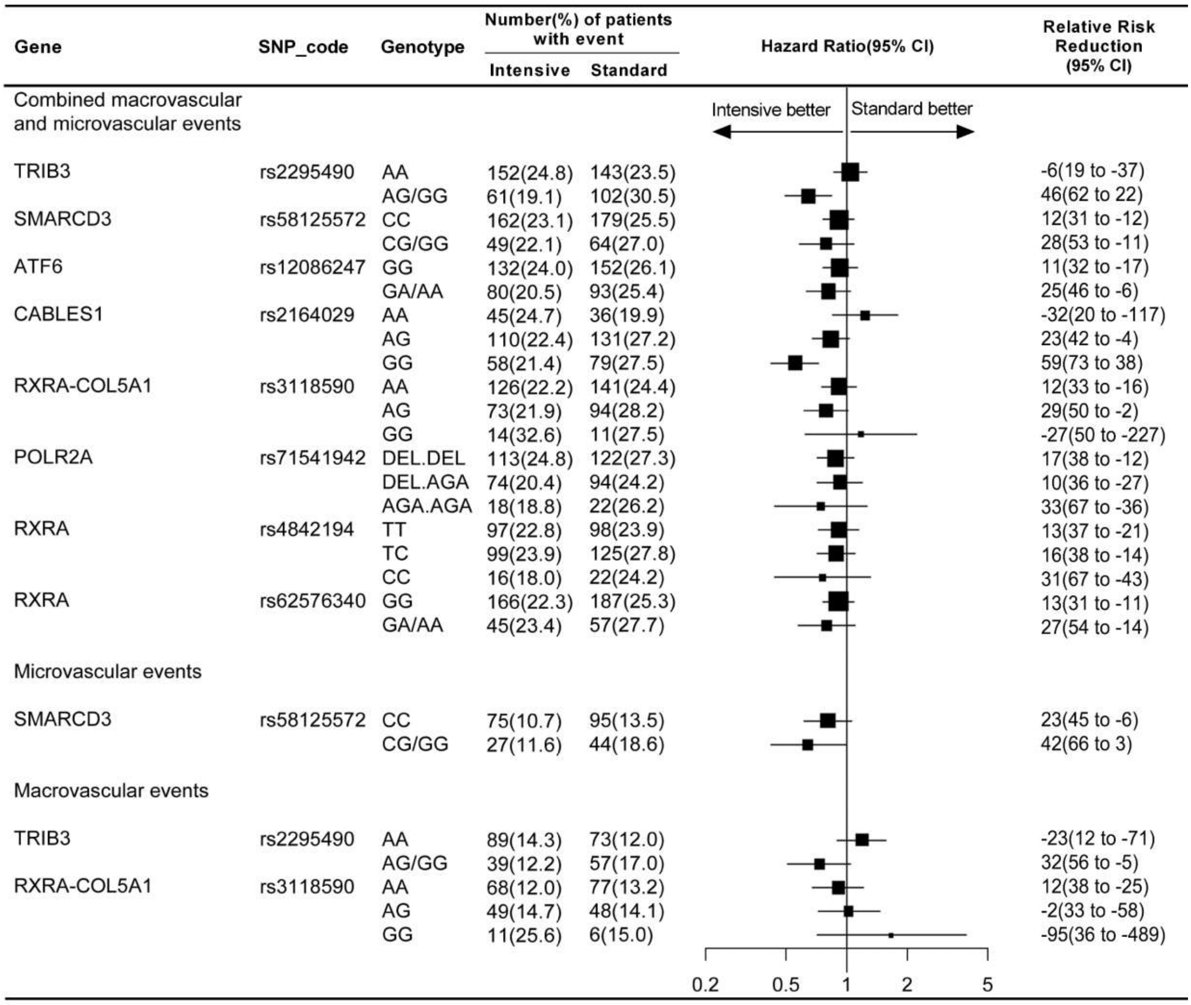

For each sub-endpoint, black squares represent point estimates, the area of the square proportional to the number of events, and horizontal lines represent 95% CI. The hazard ratios and relative risk reductions are given for intensive glucose control as compared with standard glucose control by each genotype.

Figure S2 Effect of genetic variants on major macrovascular, microvascular events or both according to blood pressure lowering intervention treatment.

Gene	SNP_code	Genotype	Number(%) of patients with event: Active	Number(%) of patients with event: Placebo	Hazard Ratio(95% CI)	Relative Risk Reduction (95% CI)
Combined macrovascular and microvascular events						
TRIB3	rs2295490	AA	139(22.8)	156(25.1)		12(32 to -15)
		AG/GG	75(22.9)	88(26.9)		19(43 to -15)
SMARCD3	rs58125572	CC	161(23.1)	180(25.5)		12(31 to -12)
		CG/GG	53(22.6)	60(25.3)		15(44 to -31)
ATF6	rs12086247	GG	125(21.8)	159(28.6)		30(47 to 9)
		GA/AA	88(24.2)	85(21.6)		-16(18 to -62)
CABLES1	rs2164029	AA	36(21.2)	45(23.3)		12(46 to -45)
		AG	114(22.6)	127(27.1)		21(41 to -5)
		GG	64(24.2)	73(24.9)		4(35 to -42)
RXRA-COL5A1	rs3118590	AA	130(22.7)	137(24.0)		7(29 to -22)
		AG	74(22.4)	93(27.7)		25(47 to -7)
		GG	10(28.6)	15(31.3)		12(66 to -129)
POLR2A	rs71541942	DEL.DEL	110(23.7)	125(28.5)		22(42 to -4)
		DEL.AGA	75(21.7)	93(23.0)		7(34 to -31)
		AGA.AGA	26(27.4)	14(16.5)		-91(8 to -296)
RXRA	rs4842194	TT	98(24.0)	97(23.9)		0(27 to -39)
		TC	101(23.3)	123(27.8)		21(42 to -7)
		CC	15(17.7)	22(24.2)		33(68 to -40)
RXRA	rs62576340	GG	166(22.9)	187(24.8)		10(29 to -14)
		GA/AA	47(22.7)	55(28.8)		27(54 to -14)
Microvascular events						
ATF6	rs12086247	GG	63(11.0)	89(11.2)		35(54 to 8)
		GA/AA	46(12.6)	44(16.0)		-15(26 to -78)

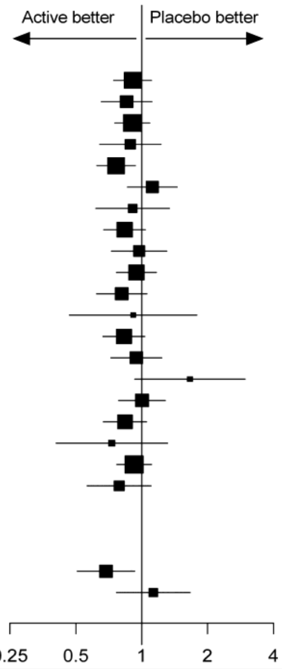

For each sub-endpoint, black squares represent point estimates, the area of the square proportional to the number of events, and horizontal lines represent 95% CI. The hazard ratios and relative risk reductions are given for intensive glucose control as compared with standard glucose control by each genotype.
